# Mitsugumin 53 Inhibits Angiogenesis Through Regulating Focal Adhesion Turnover and Tip Cell Formation

**DOI:** 10.1111/jcmm.70439

**Published:** 2025-02-24

**Authors:** Shuangshuang Yuan, Qin Yu, Tangting Chen, Tian Li, Yongjie Li, Xin Deng, Ni Chen, Jingcan You, Rong Li, Yan Liu, Youkun Zheng, Mao Luo, Hongbin Lv, Jianbo Wu, Liqun Wang

**Affiliations:** ^1^ Basic Medicine Research Innovation Center for Cardiometabolic Diseases, Ministry of Education, Luzhou Municipal Key Laboratory of Thrombosis and Vascular Biology, Laboratory for Cardiovascular Pharmacology, Department of Pharmacology, School of Pharmacy Southwest Medical University Luzhou China; ^2^ Anshun Xixiu District People's Hospital Anshun China; ^3^ Key Laboratory of Medical Electrophysiology, Ministry of Education and Medical Electrophysiological Key Laboratory of Sichuan Province, Collaborative Innovation Center for Prevention and Treatment of Cardiovascular Disease of Sichuan Province, Institute of Cardiovascular Research Southwest Medical University Luzhou China; ^4^ Metabolic Vascular Disease Key Laboratory of Sichuan Province, the Affiliated Hospital Southwest Medical University Luzhou China; ^5^ Department of Ophthalmology, the Affiliated Hospital Southwest Medical University Luzhou China

**Keywords:** angiogenesis, endocytosis, focal adhesion, mitsugumin 53, tip cell

## Abstract

Our previous studies have identified mitsugumin 53 (MG53) as a novel regulator for angiogenesis by directly entering endothelial cells and modulating focal adhesion kinase (FAK) activation, but little is known about how rhMG53 is taken up by cells and how rhMG53 mediates cell movement. In the present study, we demonstrated that the knockdown of caveolin‐1 and the clathrin inhibitor, pitstop‐2, both significantly reduced the entry of rhMG53 into endothelial cells, indicating caveolae‐dependent and clathrin‐dependent endocytosis during this process. The internalised rhMG53 remarkably inhibited the phosphorylation of FAK and the downstream signalling molecule paxillin, consequently resulting in a significant decrease in focal adhesion turnover during endothelial cell spreading and migration. Using a 3D collagen culture model, we further found that rhMG53 significantly inhibited tip cell formation and tubulogenesis. Furthermore, rhMG53 also remarkably prevented alkaline injury‐induced corneal neovascularization in vivo. Taken together, these results indicate that rhMG53 inhibits angiogenesis through regulating focal adhesion turnover and tip cell formation. This may elucidate novel molecular mechanisms involved in rhMG53 uptake and rhMG53‐modulated endothelial cell function and provide evidence for the potential utility of rhMG53 in treating diseases with excessive angiogenesis.

## 1 Introduction

MG53, a member of the tripartite motif (TRIM) family proteins, is primarily expressed in striated muscles (cardiac and skeletal muscles). Cai et al. [1] first demonstrated that MG53 in skeletal muscles can facilitate the repair of acute plasma membrane damage as an essential component in the membrane repair machinery. MG53 in cardiac muscles plays a crucial role in protecting the myocardium from ischemia/reperfusion (I/R) injury through participating in ischemic preconditioning and ischemic postconditioning [2, 3, 4]. Studies also indicate that native MG53 locates and plays important roles in non‐striated muscle tissues, such as lung epithelial cells [5, 6], inner cortex of the kidney [7, 8, 9], macrophages [10], aortic valves [11], corneal epithelial cells [12], tear film and aqueous humour [12].

Although the native MG53 is absent in a variety of tissues, interestingly, MG53 as a myokine/cardiokine can be secreted into the blood circulation from cardiac and skeletal muscles and participate in the regulation of multiple physiological and pathological processes, indicating the therapies based on its use are less likely to arouse an immune system reaction [13]. For the past few years, a rising number of researchers have confirmed the extracellular application of the recombinant human MG53 (rhMG53) to treat a range of diseases, including eccentric contraction‐ and cardiotoxin‐induced skeletal muscle injury [14], I/R‐induced myocardial infarction [2, 3, 4], I/R‐, nephrotoxin‐, cisplatin‐, contrast‐ and unilateral ureteral obstruction‐induced kidney injury [7, 8, 9] and I/R‐induced liver injury [15].

Although previous reports [13] have indicated that rhMG53 can directly bind to the extracellular domain of the insulin receptor, thereby inhibiting insulin‐mediated Akt activation, no study has yet identified the specific membrane receptor for MG53. Endocytosis is one of the key mechanisms through which extracellular rhMG53 exerts its effects in various cell types [5, 6, 7, 8, 11, 12, 16]. In our previous reports [16], we demonstrated that rhMG53 may act as a novel regulator for angiogenesis by directly entering endothelial cells, decreasing focal adhesion kinase (FAK) activation and consequently inhibiting endothelial cell migration and tube formation. However, the molecular mechanisms underlying rhMG53 entry into endothelial cells remain to be fully understood. Endocytosis is a basic process by which cells internalise a variety of extracellular materials, and multiple different endocytic modes have been identified in endothelial cells, including phagocytosis, macropinocytosis, clathrin‐dependent endocytosis, caveolae‐dependent endocytosis and combined clathrin−/caveolae‐independent processes [17, 18]. Our published data [16] showed that rhMG53 entered endothelial cells in a cholesterol‐dependent manner, indicating a caveolae‐dependent endocytosis in this process, but other pathways have not been thoroughly excluded. Therefore, the present study firstly aimed to identify the specific pathway of rhMG53 uptake into endothelial cells.

In addition, although we have demonstrated that rhMG53 may inhibit angiogenesis through regulating FAK phosphorylation [16], it still needs to be clarified how rhMG53 controls endothelial cell movement. The movement of adherent cells primarily depends on the assembly and disassembly (turnover) of focal adhesions (FAs) [19, 20]. FAs, which are small actin‐based structures that connect cells to the extracellular matrix, contain multiple proteins, including FAK and its downstream signalling paxillin. At the leading edge of the migrating cells, the activated FAK phosphorylates paxillin, leading to the interaction of these proteins with each other and with other proteins, which consequently plays crucial roles in regulating the dynamic turnover of FAs [21, 22, 23, 24]. In consideration of the inhibitory effects of rhMG53 on FAK phosphorylation reported in our previous studies, we hypothesize that MG53 may directly inhibit endothelial cell movement through inducing FA disassembly in the present study.

Angiogenesis is a complicated and multi‐stage process that involves dynamic alterations in the shapes and behaviours of endothelial cells, particularly in specialised cell types such as tip cells and stalk cells. At the forefront of a sprouting vessel, the tip cells, which have numerous filopodia, lead the vessel towards a stimulus that promotes angiogenesis. Following the tip cells, stalk cells proliferate to extend the vessel and form a lumen [25, 26, 27]. Previous studies have shown that it is feasible to assess the development of tip cells and endothelial lumen in 3D collagen matrices [28]. Therefore, the present study also investigates the effects of MG53 on endothelial tip cell formation in a 3D‐collagen culture model.

Here, we first identify the molecular mechanisms underlying rhMG53 entry into endothelial cells and show that both caveolae‐dependent and clathrin‐dependent endocytosis may contribute to rhMG53 uptake. Moreover, we have also demonstrated that rhMG53 priming significantly inhibits FA turnover and tip cell formation, which may explore the mechanisms involved in the inhibitory effects of MG53 on angiogenesis.

## 2 Materials and Methods

### 2.1 Chemicals and Reagents

Primary human umbilical vein endothelial cells (HUVECs) (#8000) and the endothelial cell medium (#1001) were purchased from ScienCell Research Laboratories (Carlsbad, CA, USA). rhMG53 was obtained from Novoprotein (Shanghai, China). Growth‐factor‐reduced Matrigel Matrix (#354230) was purchased from Corning (Corning, NY, USA). Collagen type I (#08‐115), fibronectin (FN) (#F2006), IB4 (#L2895), wortmannin (#W3144) and anti‐vinculin antibody were obtained from Merck (Darmstadt, Germany). Pitstop‐2 (ab120687), pitstop‐2 negative control (ab120688), anti‐MG53 antibody (#ab83302) used for western blotting and anti‐active integrin β1 antibody (#ab30394) were from Abcam (Cambridge, MA, USA). Anti‐MG53 antibody (#HPA023122) used for immunostaining was from Atlas Antibodies (Stockholm, Sweden). Antibodies to phosphorylated FAK^Y397^ (#3283), phosphorylated FAK^Y925^ (#3284), FAK (#3285), phosphorylated Paxillin^Y118^ (69363), Paxillin (50195), Caveolin‐1 (Cav‐1) (#3267), EEA1 (#48453), Rab7 (#95746), LAMP1 (15665), total integrin β1 (#9699), integrin β3 (#4702) and integrin α5 (#4705) were obtained from Cell Signalling Technology (Beverly, MA, USA). Antibodies to β‐actin (#AA128) and GAPDH (#AG109), HRP‐conjugated goat IgG (#A0216 and #A0208) and rhodamine‐conjugated phalloidin (#C2207) were from Beyotime Biotechnology (Shanghai, China). Methyl‐β‐cyclodextrin (MβCD) (#abs47047467) was from Absin Bioscience (Shanghai, China). Cav‐1 siRNAs and scrambled siRNA were generated by GenePharma (Shanghai, China).

### 2.2 Animals

Female C57BL/6J mice (6–8 weeks old) were purchased from Chongqing Medical University Animal Center (Chongqing China). The mice were maintained in a controlled environment (20°C–22°C, 12:12 h light/dark cycles). All procedures for animal use were approved by the Animal Care and Use Committee of Southwest Medical University (approval number: 20210923‐1).

### 2.3 Cell Culture

HUVECs were cultured in endothelial cell medium with 5% (v/v) fetal bovine serum (FBS), 1% (v/v) endothelial cell growth supplement and 1% (v/v) antibiotic solution. Cells from passages 4 to 10 were utilised.

### 2.4 siRNA‐Mediated Knockdown of Cav‐1

siRNA transfection was performed according to the protocols provided by the manufacturer. Briefly, HUVECs were allowed to grow to 60% confluence, followed by transfection with specific siRNAs and GP‐transfect‐Mate (GeenPhama) in endothelial cell medium with no FBS and no antibiotic solution for 6 h. After that, the cells were exposed to medium containing 5% FBS and 1% antibiotic solution for another 48 h before the experiments. The efficiency of siRNA‐induced Cav‐1 knockdown was confirmed by western blotting. siRNAs from GenePharma were as follows:

Scrambled siRNA: 5′‐UUCUCCGAACGUGUCACGUTT‐3′.

Cav‐1 siRNA1: 5′‐GCAGUUGUACCAUGCAUUATT‐3′.

Cav‐1 siRNA2: 5′‐GGAUCAACCAUCGCUUUAUTT‐3′.

### 2.5 Tip Cell and Tubulogenesis Assay in 3D Collagen Matrices

The detailed protocols for performing endothelial cell 3D morphogenic assays in vitro have been described in previous studies [28]. Briefly, HUVECs were incubated for 24 h with either vehicle control or rhMG53 (20 μg/mL). Cells were then collected and seeded into 3D collagen gel cultures, followed by feeding with media containing reduced serum supplement II (RSII), ascorbic acid and FGF‐2 at 40 ng/mL. The cultures were allowed to form tip cells for 24 h and to assemble a capillary network for 72 h. Then, the cultures were fixed and stained with rhodamine‐conjugated phalloidin to visualise F‐actin for use in imaging and statistical analysis.

### 2.6 Immunoblotting

Proteins were extracted from HUVECs lysed in ice‐cold RIPA lysis buffer supplemented with 1% protease and phosphatase inhibitor cocktail. Then, equal amounts of cell total proteins were separated using SDS‐PAGE, followed by transfer to a polyvinylidene fluoride membrane. In various experiments, the membranes were blocked by 5% nonfat milk at room temperature for 1 h and incubated with primary antibodies for MG53 (1:1000), Cav‐1 (1:1000), phosphorylated FAK^Y397^ (1:1000), phosphorylated FAK^Y925^ (1:500), phosphorylated Paxillin^Y118^ (1:1000), total FAK (1:1000), total Paxillin (1:1000), active integrin β1 (1:500), total integrin β1 (1:1000), integrin β3 (1:1000) and integrin α5 (1:1000) overnight at 4°C. Subsequently, the membranes were treated with HRP‐conjugated species‐specific goat IgG at room temperature for 1 h. The protein bands were then visualised using chemiluminescence, and the density of the bands was measured using ImageJ software.

### 2.7 Immunofluorescence Staining

To stain HUVECs cultured in 2D wells, the cells were fixed with 4% paraformaldehyde for 15 min at room temperature, followed by exposure to 0.5% Triton X‐100 for 15 min at 4°C. Then, the cells were blocked with 5% bovine serum albumin at 37°C for 1 h and incubated with primary antibodies against MG53 (1:50), Cav‐1 (1:50), EEA1 (1:50), RAB7 (1:50), LAMP1 (1:50) and vinculin (1:50) at 4°C overnight. After washing with PBS, the cells were incubated with Alexa 488‐ or Alexa 549‐conjugated species‐specific goat IgG (1:100) against the primary antibody applied at room temperature for 2 h. For F‐actin staining, the blocked cells were exposed to rhodamine‐conjugated phalloidin (1:100) at room temperature for 2 h. In some experiments, the nuclei were stained with 4′, 6‐diamidino‐2‐phenylindole (DAPI).

To determine tip cell formation and tubulogenesis in 3D collagen matrices, the cultures were fixed with 2% paraformaldehyde at room temperature for 2 h and treated with 0.5% Triton X‐100 at room temperature for 1 h. Then the gels were blocked for 2 h with 5% bovine serum albumin at room temperature, followed by incubation with rhodamine‐conjugated phalloidin (1:100) overnight at 4°C. Gels were then washed over several hours with PBS, and DAPI was added to visualise nuclei.

### 2.8 Cell Spreading Assay

HUVECs were incubated for 24 h with either vehicle control or rhMG53 (20 μg/mL). 24‐well plates were precoated with FN (10 μg/mL) for 1 h at 37°C. The cells were then detached and seeded onto FN‐coated 24‐well plates and exposed to FBS‐free endothelial cell medium for 15, 30, 60 and 120 min, respectively. F‐actin was stained with rhodamine‐conjugated phalloidin for use in imaging.

### 2.9 Cell Wound Healing Migration Assay Using Ibidi μ‐Dishes

Ibidi μ‐Dishes (Ibidi GmbH, Martinsried, Germany) containing the Culture‐Insert 4 well were used to perform wound healing migration. The Culture‐Insert 4 well consists of four wells, which are separated by the Culture‐Insert and used for cell culture. HUVECs were seeded in Culture‐Insert 4 Well and were allowed to grow to 90% confluence, followed by incubation with vehicle control or rhMG53 (20 μg/mL) for 24 h. At the end of the incubation, the Culture‐Insert was removed and images were acquired to obtain the initial images (0 h). Then, the cells were allowed to migrate for another 24 h, and the migration area was calculated and quantitatively analysed.

### 2.10 Cell Migration Assay Using Transwell Chambers

Cell migration was also performed using transwell chambers with an 8.0 μm‐sized porous membrane. HUVECs were incubated for 24 h with either vehicle control or rhMG53 (20 μg/mL). The cells were then detached and added to the upper chambers containing porous filters. After 24 h, the cells were fixed with 4% paraformaldehyde for 10 min at room temperature and stained with 0.5% crystal violet for 15 min. Then, cells remaining in the upper chambers were removed with a cotton swab, and the cells that have migrated to the lower chambers were photographed and counted.

### 2.11 Tube Formation Assay

HUVECs were incubated for 24 h with either vehicle control or rhMG53 (20 μg/mL). 24‐well plates were precoated with growth‐factor‐reduced Matrigel Matrix (250 μL/per well) for 30 min at 37°C. The cells were then detached, seeded onto Matrigel and exposed to endothelial cell medium containing 1% FBS for 18 h. Tube formation was photographed, and the total tube length was quantified using ImageJ software.

### 2.12 FA Formation Assay

To determine FA formation during cell spreading, HUVECs were incubated for 24 h with either vehicle control or rhMG53 (20 μg/mL). Then, the cells were detached, seeded onto FN (10 μg/mL)‐coated petri dishes and exposed to FBS‐free endothelial cell medium for 2 h. After that, vinculin was immunofluorescently stained to label FAs, and the number of FAs was calculated.

To determine focal adhesion formation during cell migration, HUVECs were seeded onto 24‐well plates and allowed to grow to 90% confluence, followed by incubation for 24 h with either vehicle control or rhMG53 (20 μg/mL). Then, the cells were scratched with a 200 μL pipette tip to make a wound healing and allowed to migrate for additional 0, 2 and 4 h. After immunostaining for vinculin, the number of focal adhesions at the edge of the scratch was counted, and the ratio of focal adhesion number normalised to the edge length was quantitatively analysed.

### 2.13 Animal Models

Corneal wound healing models [12] were used in this study. A 2 mm disk of filter paper soaked in NaOH (1 mol/L) was applied to the right cornea of each mouse for 40 s to induce injury. Then, the injured corneas were topically applied PBS or rhMG53 (2 μg/mL) for 30 mL/eye twice daily for a total of 7 days. At 7 days following treatment, animals were euthanized, and globes were enucleated. In all subsequent histologic analyses, the individual was masked to the treatment.

### 2.14 Cornea Whole‐Mount Immunostaining

Mouse cornea whole‐mount immunostaining was performed as previously described. In brief, the eyes were harvested and immediately fixed with 4% paraformaldehyde overnight at 4°C, followed by incubation with 0.5% Triton X‐100 for 2 h at room temperature. Then, the eyes were washed three times with ice‐cold PBS, and the corneas were dissected and cut into four radial incisions under a stereomicroscope. After that, the corneas were washed and incubated with endothelial marker isolectin B4 (1:100) at 4°C overnight. Following thorough washing, the corneas were transferred to the glass slide, flat‐mounted and sealed under a glass coverslip in mounting media.

### 2.15 Statistical Analysis

Student's *t* test or one‐way ANOVA, followed by post hoc comparison was performed to analyse the results. All of the data are shown as mean ± standard deviation (SD). A *p* value of < 0.05 was considered statistically significant.

## 3 Results

### 3.1 rhMG53 Enters Endothelial Cells Through Caveolae‐Dependent and Clathrin‐Dependent Endocytosis

**FIGURE 1:**
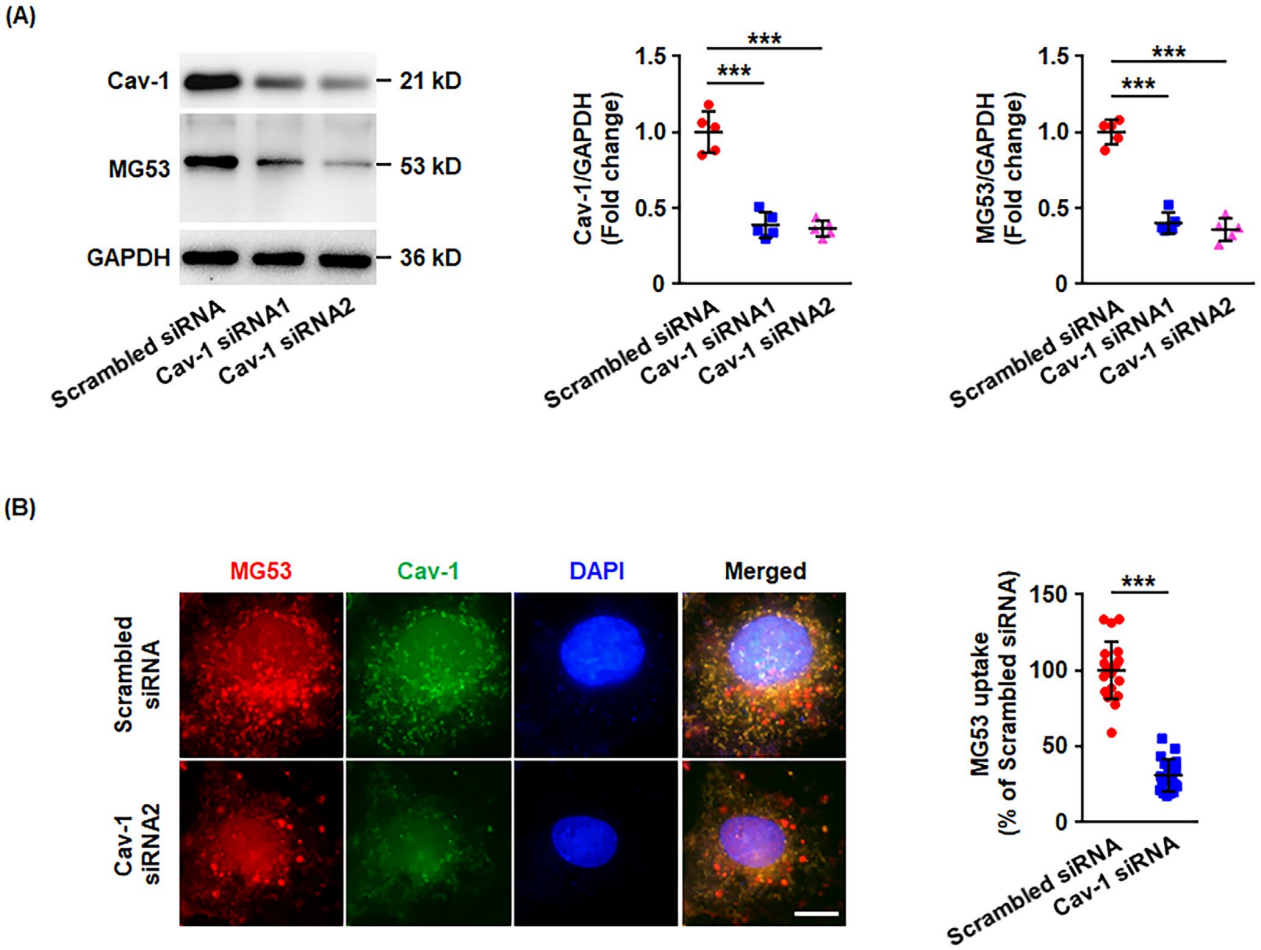
Cav‐1 is required for the uptake of rhMG53 into endothelial cells. HUVECs were transfected with scrambled or Cav‐1 siRNA, followed by stimulation with rhMG53 (20 μg/mL) for 60 min. (A) Total proteins were collected and western blotting was carried out to evaluate Cav‐1 expression and intracellular MG53. Representative images are shown on the left. The densitometric analysis of Cav‐1 or MG53 normalised to GAPDH was performed (*n* = 5 biological replicates). (B) HUVECs were fixed and then immunohistochemical staining was performed for MG53 (red) and Cav‐1 (green). DAPI‐stained nuclei are shown in blue. Representative images from four independent experiments are shown. The scale bar is 10 μm. The average fluorescent intensity of MG53 per cell was determined (*n* = 20 fields of view per group, from 4 independent experiments). For the above, data are presented as mean ± SD. ***p < 0.001 (one‐way ANOVA with Tukey's multiple comparisons in A, two‐tailed unpaired Student's *t* test in B).

Our previous studies have indicated the direct entry of rhMG53 into endothelial cells [16]. To explore the specific mechanism of rhMG53 uptake, HUVECs were pretreated with the cholesterol inhibitor, MβCD, followed by exposure to rhMG53. In agreement with our previous data [16], both the western blotting and immunostaining results demonstrated that the depletion of cholesterol by MβCD significantly decreased the uptake of rhMG53 by endothelial cells (Figure S1), indicating a caveolae‐dependent endocytosis in this process. In endothelial cells, Cav‐1 is one of the major membrane proteins essential for caveolae formation [9, 29]. Therefore, the effects of Cav‐1 on rhMG53 cellular uptake were also determined. Consistent with the experiments involving MβCD, the knockdown of Cav‐1 by siRNAs remarkably inhibited the entry of rhMG53 into endothelial cells (Figure 1). All the results suggest that rhMG53 enters endothelial cells in a caveolae‐dependent manner. Furthermore, the merging of MG53 and Cav‐1 staining revealed that the majority of the rhMG53 fluorescence co‐localised with Cav‐1, while a small part of rhMG53 did not interact with Cav‐1 (Figure 1B), indicating that other endocytosis mechanisms may be involved in rhMG53 uptake.

**FIGURE 2:**
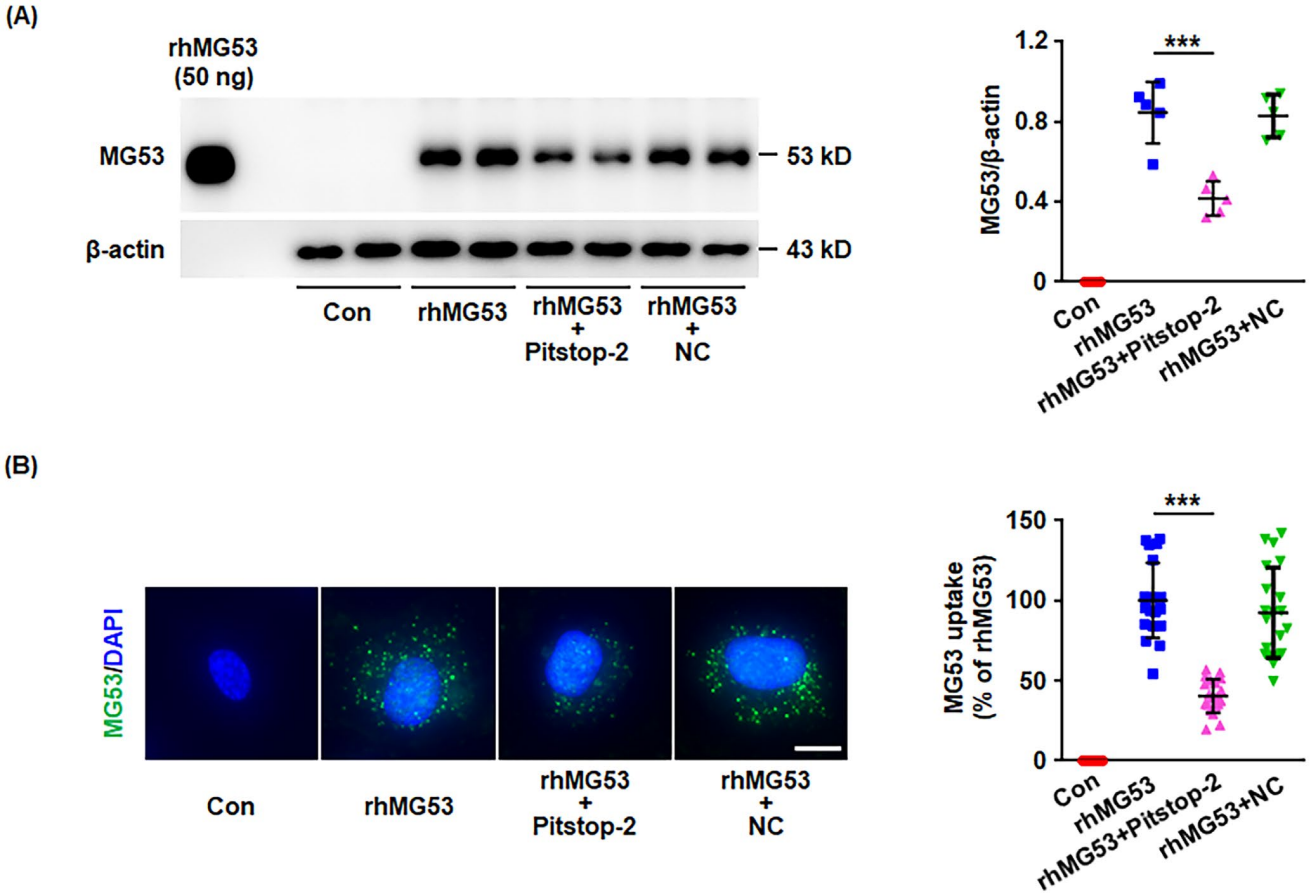
Pitstop‐2 decreases rhMG53 uptake into endothelial cells. HUVECs were pretreated with pitstop‐2 (5 μM) or the negative control (NC, 5 μM) for pitstop‐2 for 30 min, followed by stimulation with rhMG53 (20 μg/mL) for 60 min. (A) Cell lysates were harvested and intracellular MG53 was detected by western blotting. Representative images are shown on the left. The densitometric analysis of MG53 normalised to β‐Actin was performed (*n* = 5 biological replicates). (B) HUVECs were fixed and incubated with a primary anti‐MG53 antibody, followed by immunostaining using a FITC‐conjugated secondary antibody (green). DAPI‐stained nuclei are shown in blue. Representative images from four independent experiments are shown. The scale bar is 10 μm. The average fluorescence intensity of MG53 per cell was determined (*n* = 20 fields of view per group, from four independent experiments). For the above, data are presented as mean ± SD. ***p < 0.001 (one‐way ANOVA with Tukey's multiple comparisons).

Therefore, clathrin‐dependent endocytosis and macropinocytosis were further investigated. HUVECs were pretreated with the clathrin inhibitor, pitstop‐2, or the macropinocytosis inhibitor, wortmannin, followed by stimulation with rhMG53. The western blotting and immunostaining results showed that pitstop‐2 significantly inhibited the entry of rhMG53 into endothelial cells (Figure 2), suggesting that the cellular uptake of rhMG53 may be partly through clathrin‐dependent endocytosis. However, the macropinocytosis inhibitor, wortmannin, had little effect on rhMG53 uptake (Figure S2).

### 3.2 Internalised rhMG53 Is Targeted to the Endolysosomal Compartment

**FIGURE 3:**
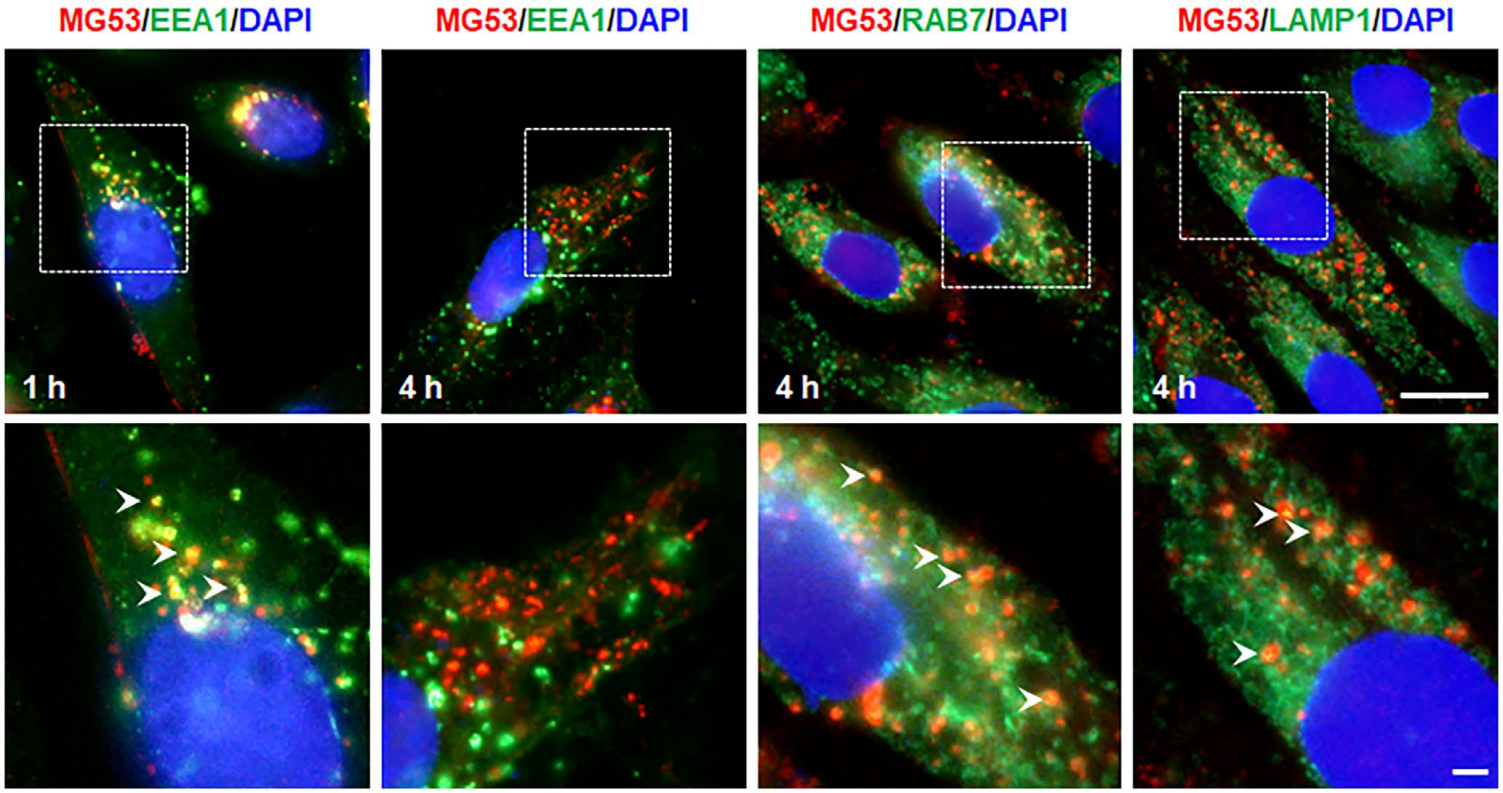
Internalised rhMG53 is targeted to the endosome and lysosome. HUVECs were stimulated with rhMG53 (20 μg/mL) for 1 h or 4 h. The co‐localization of MG53 (red) with the early endosomal marker EEA1 (green), with the late endosomal marker Rab7 (green) and with the lysosomal marker LAMP1 was identified by immunostaining. DAPI‐stained nuclei are shown in blue. Representative images from four independent experiments are shown. Arrowheads indicate examples of co‐localization. The scale bar is 10 μm.

To investigate the destination of the internalised rhMG53, co‐localisation experiments were performed using markers for endosomal and lysosomal compartments. MG53 puncta were positive for EEA1 (early endosomes) 1 h after initiating endocytosis. At the 4 h time point, rhMG53 partially co‐localised with RAB7 (late endosomes) and partially with LAMP (lysosomes) (Figure 3). All the data indicate that internalised rhMG53 is targeted to the endolysosomal compartment.

### 3.3 rhMG53 Inhibits the Activation of the FAK‐Paxillin Signalling Pathway

**FIGURE 4:**
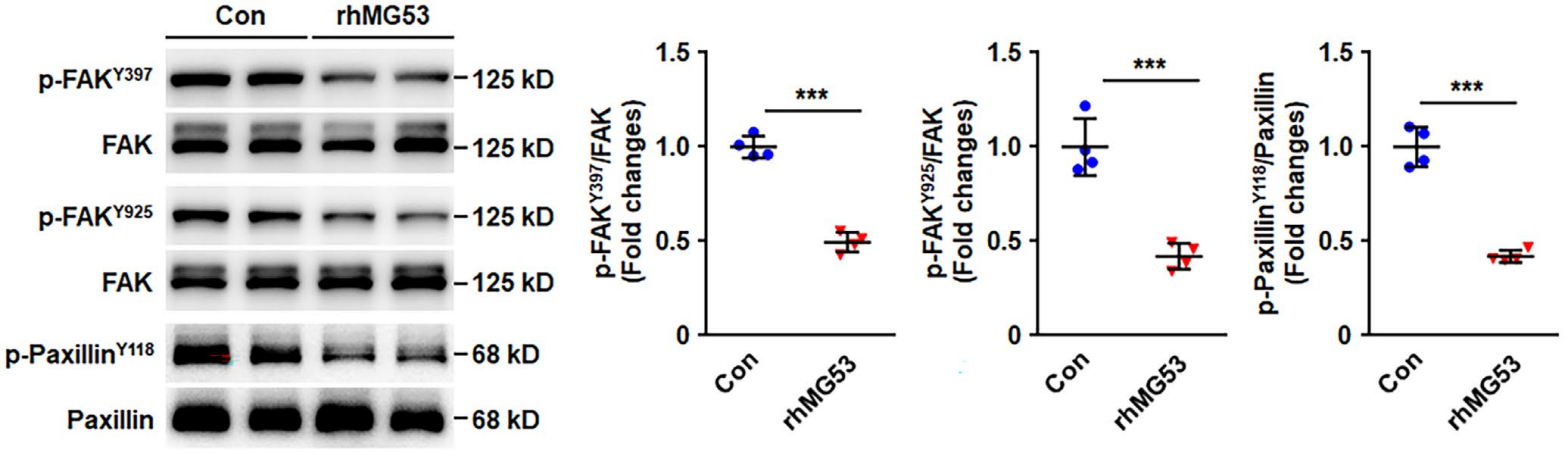
rhMG53 inhibits FAK‐paxillin signalling pathway activation. HUVECs were incubated for 24 h with either vehicle control or rhMG53 (20 μg/mL), and then the cell lysates were prepared. Phosphorylation of FAK^Y397^, FAK^Y925^ and paxillin^Y118^, and total FAK and paxillin were analysed by western blotting. Representative images from four independent experiments are shown. The densitometric analysis of phosphorylation of FAK and paxillin normalised to total FAK and paxillin was carried out. All data shown is presented as mean ± SD. ***p < 0.001 (two‐tailed unpaired Student's *t* test).

To further investigate the effects of internalised rhMG53 on endothelial cell physiological responses, HUVECs were exposed to either vehicle control or rhMG53 for 24 h, which remarkably increased intracellular MG53 (Figure S3). Then, the migration and tube formation of HUVECs were determined, and the results demonstrated that rhMG53 significantly decreased endothelial cell migration number, migration area and tube length, indicating reduced movement ability in HUVECs primed with rhMG53 (Figure S4). To clarify the molecular mechanisms involved in the inhibitory effects of rhMG53 priming on endothelial cell movement, the activation of the FAK–paxillin signalling pathway was measured. Consistent with our published data [16], rhMG53 significantly inhibited the phosphorylation of FAK at Y397. Moreover, rhMG53 also remarkably reduced the activation of FAK at Y925 and inhibited the phosphorylation of the downstream signalling molecule, paxillin (Figure 4). FAK‐paxillin signalling plays crucial roles in cell movement through regulating the dynamic turnover of FAs. Therefore, the effects of rhMG53 on FA turnover would be further measured.

### 3.4 rhMG53 Inhibits FA Turnover During Cell Migration

**FIGURE 5:**
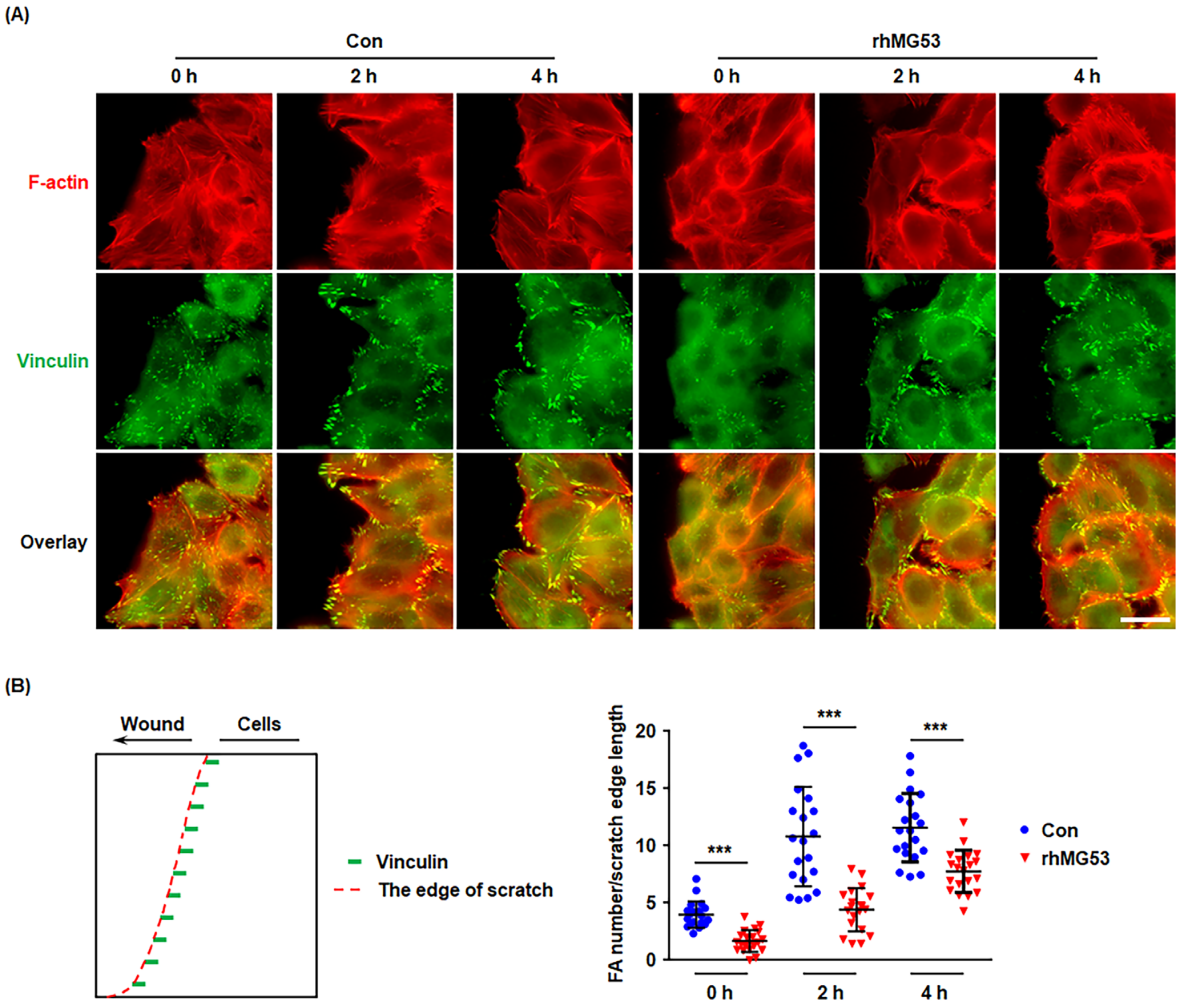
rhMG53 Inhibits FA turnover during cell migration. (A) HUVECs were allowed to grow to 90% confluence and incubated for 24 h with either vehicle control or rhMG53 (20 μg/mL). Afterward, the cells were scratched with a 200 μL pipette tip and allowed to migrate for an additional 0, 2 and 4 h, followed by immunostaining for F‐Actin (red) and vinculin (green). Representative images from four independent experiments are shown. The scale bar is 20 μm. (B) The number of FAs at the edge of the scratch was counted and the length of the scratch edge was measured. Then the ratio of FA number normalised to the edge length was quantitatively analysed (*n* = 20 fields of view per group, from four independent experiments). Data are presented as mean ± SD. ***p < 0.001 (two‐tailed unpaired Student's *t* test).

To investigate FA turnover during cell migration, HUVECs grown to 90% confluence were incubated for 24 h with either vehicle control or rhMG53 (20 μg/mL), followed by creating a wound healing. After migration for additional 0, 2 and 4 h, the formation of FAs was detected by immunostaining for vinculin. At the time of 0 h after scratching, few FAs were visualised at the edge of the wound, while FAs gradually formed with the migration of endothelial cells (Figure 5A). More importantly, during wound healing, the number of FAs at the edge of the scratch was significantly lower in cells primed with rhMG53 than in unprimed cells, indicating that rhMG53 inhibits FA turnover (Figure 5 and Figure S5).

### 3.5 rhMG53 Inhibits FA Turnover During Cell Spreading

**FIGURE 6:**
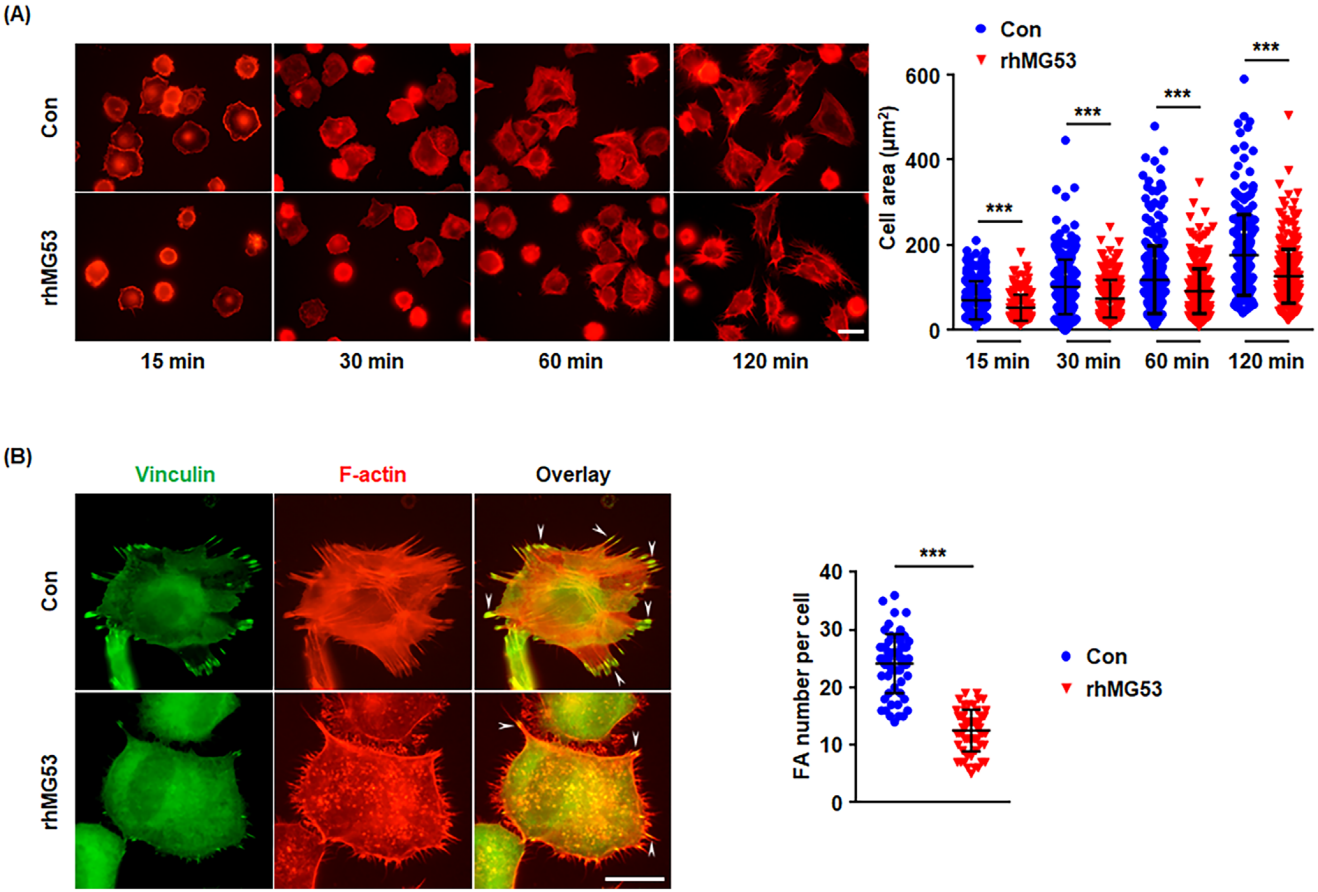
rhMG53 Inhibits endothelial cell spreading and FA turnover during cell spreading. HUVECs were incubated for 24 h with either vehicle control or rhMG53 (20 μg/mL). (A) The cells were then detached and seeded onto FN (10 μg/mL)‐coated 24‐well plates for 15, 30, 60 and 120 min, respectively. F‐Actin was stained with rhodamine‐conjugated phalloidin and representative images from 4 independent experiments are shown. The scale bar is 20 μm. The cell areas were measured, and quantitative assessment of four independent experiments was performed (*n* > 200 cells per group, from four independent experiments). (B) HUVECs were seeded onto FN (10 μg/mL) for 2 h, followed by staining for F‐Actin with rhodamine‐conjugated phalloidin and an antibody recognising vinculin. Representative images from 4 independent experiments are shown and the scale bar is 10 μm. The number of FAs (arrowheads) was calculated and quantitatively analysed. For the above, data are presented as mean ± SD. ***p < 0.001 (two‐tailed unpaired Student's *t* test).

We also turned our attention to rhMG53‐mediated FA turnover during endothelial cell spreading, which is important in the early stages of angiogenesis. rhMG53‐treated or control HUVECs were seeded onto FN‐coated 24‐well plates and allowed to spread for 15, 30, 60 and 120 min. The areas of rhMG53‐treated cells were remarkably lower than those of control cells (Figure 6A), suggesting rhMG53 inhibits endothelial cell spreading. Vinculin was then immunostained, and results showed that rhMG53 also significantly decreased FA number during cell spreading (Figure 6B).

Given the critical roles of integrins, particularly integrin β1 in FA formation and angiogenesis, we also determined the expression and activation of integrins. Although no change was detected in the expression levels of total integrin α5, integrin β3 and integrin β1 (Figure S6 and Figure S7), the active form of integrin β1 significantly decreased in rhMG53‐treated endothelial cells (Figure S7), indicating that rhMG53‐regulated FA turnover may be associated with the activation of integrin β1.

### 3.6 rhMG53 Inhibits Tip Cell Formation and Tubulogenesis in 3D Collagen Matrices

**FIGURE 7:**
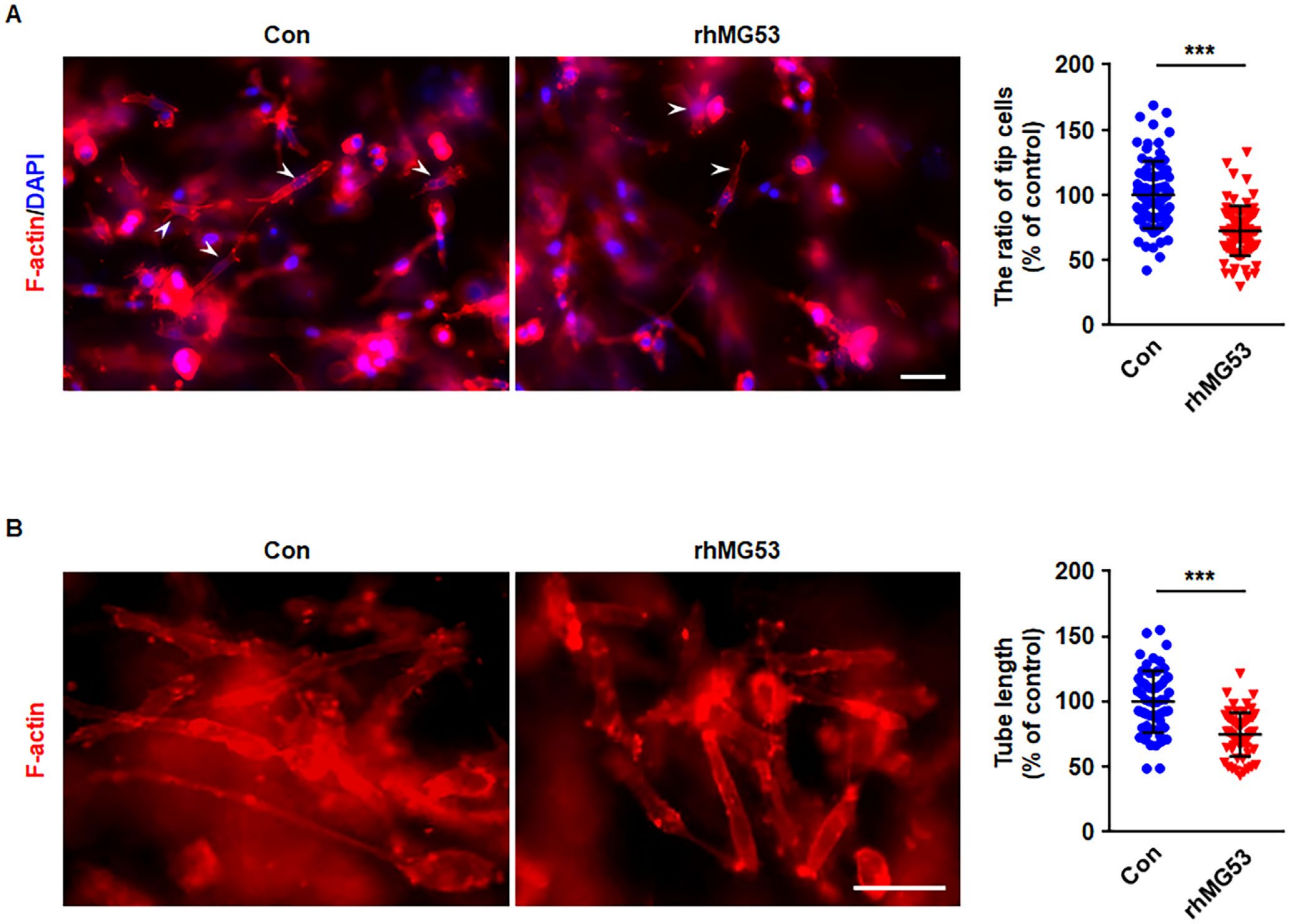
rhMG53 Inhibits tip cell formation and tubulogenesis in 3D collagen matrices. HUVECs were incubated for 24 h with either vehicle control or rhMG53 (20 μg/mL). Cells are then collected and seeded into 3D collagen gel cultures, followed by feeding with media containing reduced serum supplement II (RSII), ascorbic acid and FGF‐2 at 40 ng/mL. (A) After 24 h, the cultures were fixed with 4% paraformaldehyde and stained with rhodamine‐conjugated phalloidin to visualise F‐Actin (red). DAPI‐stained nuclei are shown in blue. Representative images from four independent experiments are shown. The scale bar is 50 μm. The number of tip cells was calculated and quantitatively analysed (*n* = 80 fields of view per group, from four independent experiments). (B) Cultures were allowed to assemble into capillary networks for 72 h, and then the cultures were fixed in 4% paraformaldehyde and stained with rhodamine‐conjugated phalloidin. Representative images from four independent experiments are shown. The scale bar is 50 μm. The length of the tube was measured and quantitatively analysed (*n* > 50 fields of view per group, from four independent experiments). For the above, data are presented as mean ± SD. ***p < 0.001 (two‐tailed unpaired Student's *t* test).

It is well known that endothelial tip cells are key stimulators of branching tube networks. Therefore, the effects of rhMG53 on tip cell generation were further investigated using a 3D collagen cell culture model. rhMG53‐treated or control HUVECs were seeded into 3D collagen gel cultures, and the results demonstrated that rhMG53 resulted in significant decreases in tip cells that were observed by 24 h of culture (Figure 7A). The observed tip cells grown in the collagen gel cultures displayed the features of tip cells in vivo with marked filopodial extensions (Figure S8A), while cells grown on the bottom of the collagen gel showed 2D cell morphology (Figure S8B). Furthermore, after 72 h culture, cells grown in the collagen gel cultures showed clear tubulogenesis (Figure S8C), and rhMG53 remarkably decreased the length of tube formation (Figure 7B). Taken together, these data indicate that rhMG53 prevents tip cell formation and tubulogenesis in 3D collagen matrices, which may contribute to the inhibitory effects of rhMG53 on angiogenesis.

### 3.7 rhMG53 Reduces Corneal Neovascularisation in a Mouse Corneal Injury Model

**FIGURE 8:**
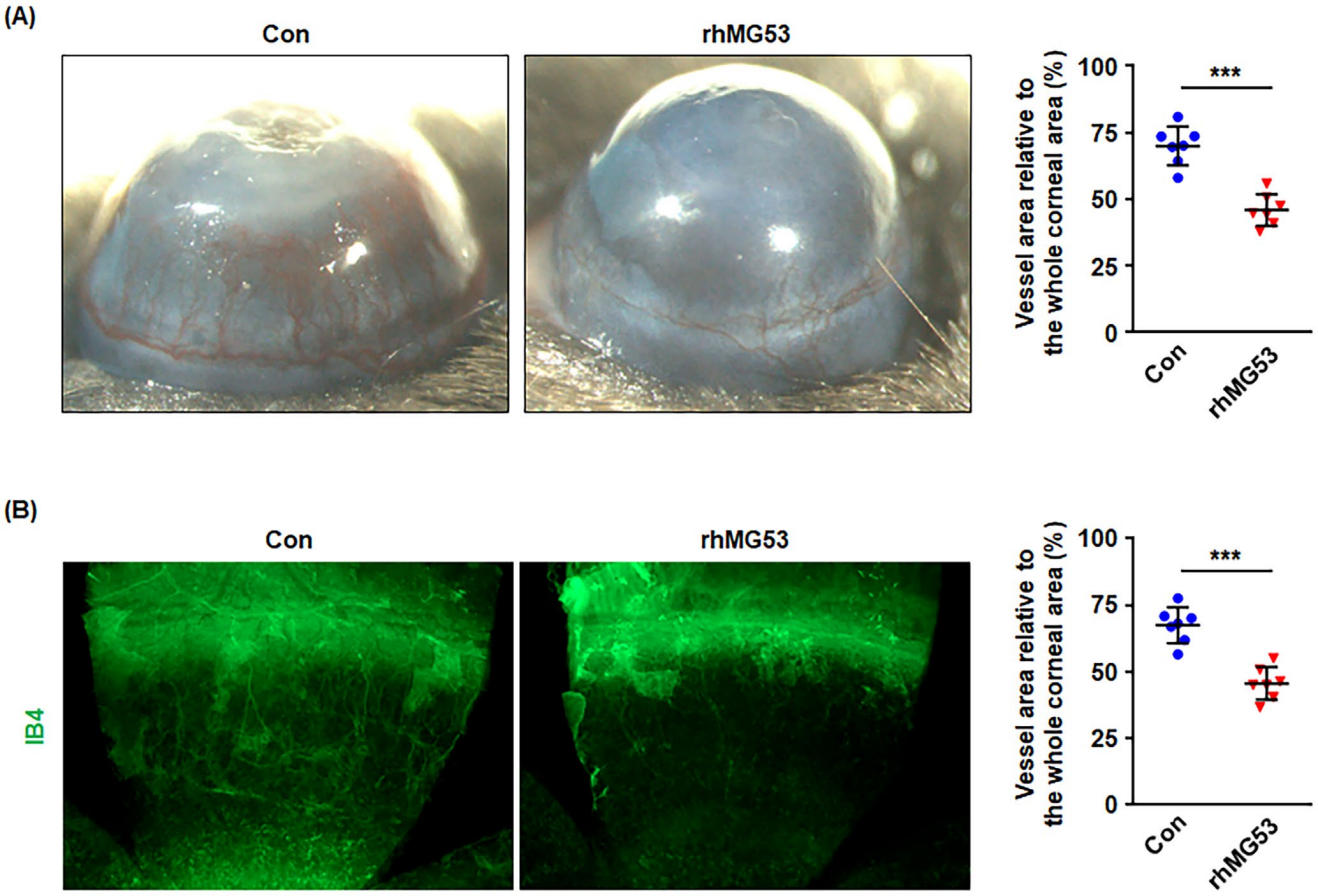
rhMG53 inhibits alkaline injury‐induced corneal angiogenesis. The corneas were exposed to a 2 mm disk of filter paper soaked in NaOH (1 mol/L) for 40 s to induce injury, followed by topically incubation with PBS or rhMG53 (2 μg/mL) for 30 mL/eye twice daily for a total of 7 days. (A) Images were taken to show the lateral views of each eye and the ratio of corneal vessel area relative to the whole corneal area was calculated. (B) Mouse corneal neovascularization was observed by whole‐cornea immunofluorescence staining for IB4 (green). Intensity of IB4 signal was used as index of vascularization of the cornea at 7 days post–alkaline injury. For the above, data are presented as mean ± SD. ***p < 0.001 (two‐tailed unpaired Student's *t* test).

Our previous studies have demonstrated that exogenous rhMG53 significantly decreased retinal angiogenesis in postnatal mice [16]. To further confirm the inhibitory effects of rhMG53 on angiogenesis in other animal models, rhMG53 was topically applied to alkaline‐induced corneas. Seven days post‐alkaline injury, the control corneas developed excessive revascularization, whereas the addition of rhMG53 significantly prevented excess corneal vascularization (Figure 8A). Furthermore, immunofluorescent staining for IB4, a specific marker for endothelial cells, showed that IB4 staining in the rhMG53‐treated corneas was remarkably less than that in control corneas (Figure 8B). The data presented here suggest that rhMG53 inhibits alkaline injury‐induced corneal neovascularization, which further supports the physiological roles for MG53 in preventing angiogenesis in vivo.

## 4 Discussion

Our previous studies have shown that rhMG53 inhibits angiogenesis through mechanisms that involve entering endothelial cells, decreasing FAK phosphorylation and consequently reducing cell migration and tube formation [16]. To further explore the molecular mechanisms underlying the regulation of rhMG53 on endothelial cells, the present study investigated how rhMG53 enters cells and how rhMG53 controls cell movement. Moreover, using a 3D‐collagen culture model, the effects of rhMG53 on tip cell formation were also determined, providing more evidence to explain how rhMG53 regulates angiogenesis.

Previous studies and our experiments involving the cholesterol inhibitor, MβCD demonstrated the crucial role of cholesterol in rhMG53 uptake [12, 16, 30]. Consistent with these results, our present data identified a co‐localization between rhMG53 and Cav‐1, the major protein component of plasma membrane lipid raft caveolae [31], and further displayed that knockdown of Cav‐1 significantly prevented the entry of rhMG53 into HUVECs, indicating that caveolae‐dependent endocytosis may be involved in rhMG53 uptake. In agreement with our results, prior reports also showed the direct interaction between endogenous MG53 and Cav‐1 in alveolar epithelial cells [6, 32]. Interestingly, a published study indicated a caveolar component, polymerase I and transcript release factor, as an obligatory docking protein for MG53 during membrane repair [33], which further supports the critical role of caveolae in MG53‐mediated biological responses. One of the major physiological functions of caveolae is to regulate clathrin‐independent endocytosis [34]. However, our present research also showed that the uptake of rhMG53 by endothelial cells partly depended on clathrin‐mediated endocytosis. In contrast to this result, most published reports focusing on cholesterol have revealed that cholesterol is required for rhMG53 uptake [12, 16, 30], indicating a clathrin‐independent endocytosis in this process. However, other studies also displayed that some ligands or receptors could be endocytosed by both caveolae‐ and clathrin‐dependent pathways [35, 36, 37], which partially supports our findings. Further research is necessary to confirm the role of clathrin‐mediated endocytosis in the uptake of rhMG53 across various cell types.

FAK and its associated signalling pathways play crucial roles in endothelial cell adhesion, migration and tube formation by participating in signal transduction triggered by integrins and other cell surface receptors [38, 39]. Our prior [16] and current data displayed the inhibitory effects of rhMG53 on FAK autophosphorylation at Y397. The present results also demonstrated that rhMG53 dramatically reduced phosphorylation of FAK at Y925 and inhibited the activation of paxillin, the downstream signalling molecule of FAK, further supporting the blockade of FAK activity by rhMG53. In agreement with these data, previous studies have shown that the phosphorylation of Y397 generates a binding site for Src, which triggers the phosphorylation of other sites on FAK and ultimately leads to its full activation [40, 41]. Moreover, Y397 phosphorylation induces an interaction between Src and FAK, which, in turn, through its focal adhesion targeting domain, cross‐reacts with Y118 of paxillin and results in paxillin activation [42]. Our previous results have verified that rhMG53 uncouples FAK‐Src crosstalk [16] and multiple published studies also displayed paxillin inactivation induced by FAK inhibition [43, 44, 45]. On the other hand, although no research has investigated the molecular mechanisms underlying rhMG53‐induced FAK inactivation, previous studies, including those of our group, have demonstrated the direct interaction between MG53 and FAK [16, 46]. Specifically, the binding of MG53 to the 4.1 protein/ezrin/radixin/moesin (FERM) and kinase domains of FAK [46], both of which are critical for FAK activity, may contribute to rhMG53‐mediated FAK dysfunction.

The significance of FAK and paxillin has been demonstrated in cell movement and the dynamic assembly and disassembly of FAs [21, 22, 23, 24]. Consistent with the inhibitory effects of rhMG53 on FAK‐paxillin signalling pathways, our experiments involving measurements of endothelial cell physiological responses showed that rhMG53 significantly inhibited FA turnover during cell spreading and migration. As a consequence, rhMG53 primed HUVECs yielded remarkably decreased cell migration and tube formation compared to unprimed cells. Previous studies demonstrated that FAK–paxillin signalling was critical for both vascular endothelial growth factor and angiopoietin‐1‐induced angiogenesis [47, 48], which partly supports the findings in the present study. Furthermore, in agreement with our findings, published data also showed that the blockade of FAK by some inhibitors decreased paxillin phosphorylation and FA turnover and consequently inhibited cell migration [49, 50, 51], which indicates the potential utility of rhMG53 as a FAK inhibitor.

In agreement with previous studies that demonstrated the critical role of integrin β1 in FA formation and cell migration [52, 53], our results also indicated that rhMG53 induced a significant decrease in the active form of integrin β1. Integrin β1 can localise both intracellularly and at the plasma membrane, and in fact, the active integrin β1 appears to be predominantly cytoplasmic [52, 54]. More interestingly, prior research has shown that only active β1 localises to RAB7‐positive compartments [52]. Our current findings demonstrated the co‐localisation of rhMG53 with RAB7, suggesting an interaction between active integrin β1 and rhMG53 in late endosomes. Given the significance of active integrin β1 in the activation of the FAK signalling pathway [55, 56], the inhibitory effects of rhMG53 on FAK phosphorylation may be associated with the rhMG53‐induced decrease in active β1.

Consistent with the cell culture results, our experiments involving the 3D collagen culture model also displayed that rhMG53 treatment induced a significant decrease in tip cell formation and tubulogenesis. Previous studies have demonstrated the crucial roles of FAK‐paxillin signalling activation in matrix stiffness‐ and endothelial neuropilin‐2‐modulated tip cell formation [57, 58]. Considering our results and the published data, the inhibitory effects of rhMG53 on tip cells may also depend on rhMG53‐regulated FAK‐paxillin signalling pathways. More meaningfully, the corneal neovascularization assay also showed that rhMG53 significantly prevented alkaline injury‐induced angiogenesis in vivo. In agreement with our results, Ma and colleagues have indicated that the *mg53* ^−/−^ mice yield more corneal neovascularization after alkaline injury compared to the wild type literatures [12] and exogenous application of rhMG53 remarkably decreased corneal angiogenesis in *db/db* mice [59].

However, some limitations need to be further studied. Firstly, live imaging of FA dynamics is required to really distinguish the effects of rhMG53 on FA turnover. Furthermore, our in vivo experiments did not confirm rhMG53‐induced inactivation of FAK‐paxillin signalling. Nevertheless, our corneal neovascularization assay complements and supports the significance of our in vitro experiments, which demonstrated that rhMG53 inhibits angiogenesis via regulating FA turnover and tip cell formation.

**FIGURE 9:**
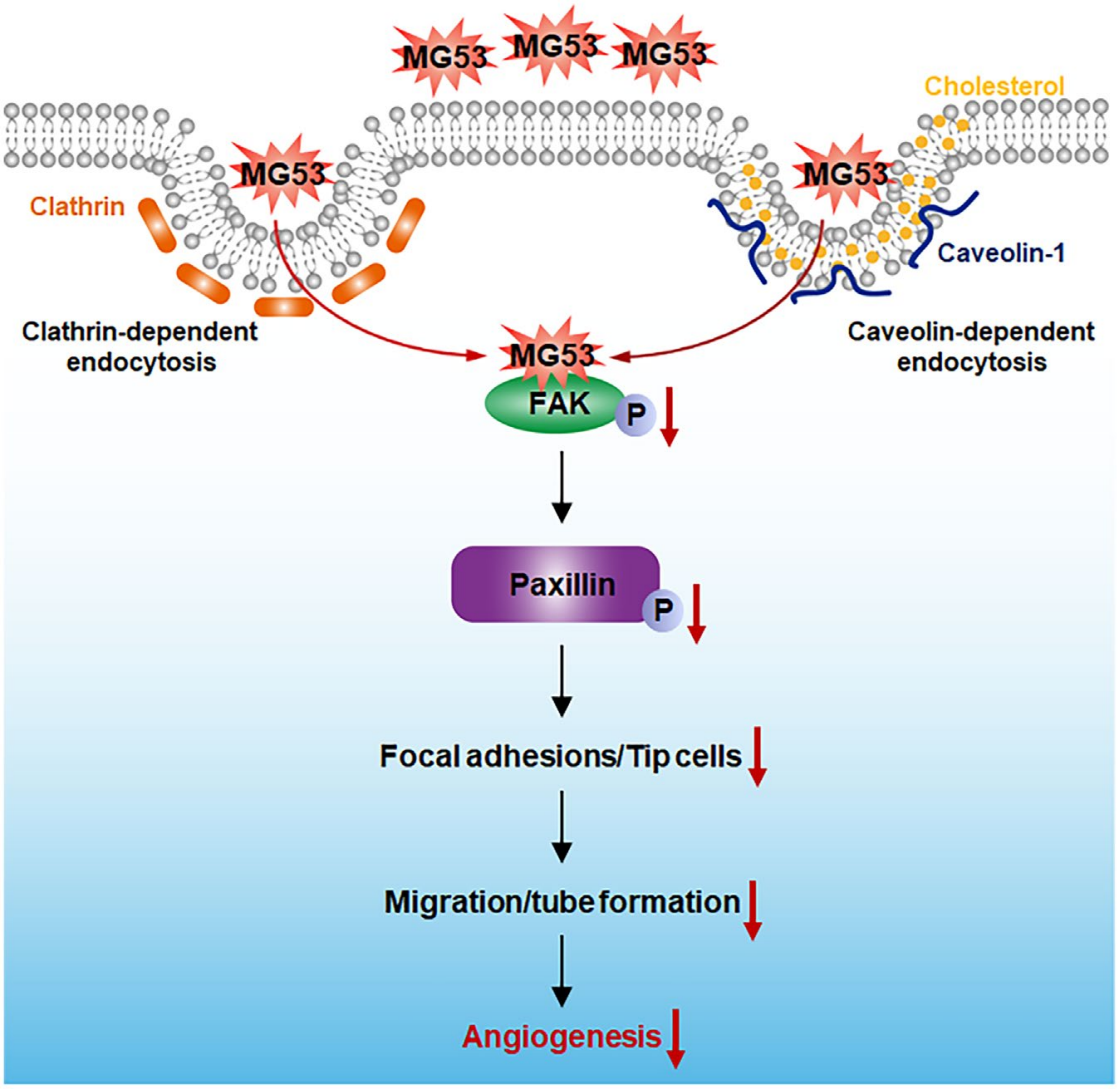
Schematic diagrams representing the molecular mechanisms underlying rhMG53‐regulated angiogenesis. rhMG53 enters endothelial cells via caveolae‐dependent and clathrin‐dependent endocytosis and then reduces FAK‐paxillin signalling activation, leading to decreased FA turnover and tip cell formation, ultimately inhibiting angiogenesis.

The present study summarises that rhMG53 enters endothelial cells via caveolae‐dependent and clathrin‐dependent endocytosis, and then reduces FAK‐paxillin signalling activation, leading to decreased FA turnover and tip cell formation, ultimately inhibiting angiogenesis (Figure 9). These findings advance our understanding of rhMG53 in modulating endothelial functions and provide evidence for the potential use of rhMG53 to treat diseases with excessive angiogenesis.

## Author Contributions

**Shuangshuang Yuan:** investigation (lead), methodology (equal), visualization (lead), writing – original draft (equal). **Qin Yu:** investigation (equal), methodology (equal), visualization (equal), writing – original draft (equal). **Tangting Chen:** funding acquisition (supporting), investigation (equal), methodology (equal), resources (equal), writing – original draft (equal). **Tian Li:** methodology (lead), resources (lead). **Yongjie Li:** methodology (equal). **Xin Deng:** methodology (equal). **Ni Chen:** methodology (equal). **Jingcan You:** methodology (equal). **Rong Li:** formal analysis (equal), methodology (equal). **Yan Liu:** methodology (equal). **Youkun Zheng:** formal analysis (equal), resources (equal). **Mao Luo:** formal analysis (equal), project administration (supporting). **Hongbin Lv:** supervision (equal), writing – review and editing (equal). **Jianbo Wu:** project administration (equal), supervision (equal), writing – review and editing (equal). **Liqun Wang:** funding acquisition (lead), investigation (supporting), project administration (lead), supervision (lead), writing – review and editing (lead).

## Conflicts of Interest

The authors declare no conflicts of interest.

## Supporting information


**FIGURE S1.** Cholesterol is required for the uptake of rhMG53 into endothelial cells.
**FIGURE S2.** Wortmannin does not inhibit the uptake of rhMG53 into endothelial cells.
**FIGURE S3.** rhMG53 induces the increase of intracellular MG53.
**FIGURE S4.** rhMG53 inhibits endothelial cell migration and tube formation.
**FIGURE S5.** The changes of vinculin fluorescence intensity at the edge of the scratch.
**FIGURE S6.** rhMG53 has no effect on the expression of integrins.
**FIGURE S7.** rhMG53 decreases the activation of integrin β1.
**FIGURE S8.** Endothelial cells cultured in 3D collagen gel cultures.

## Data Availability

The data supporting this study's findings are available from the corresponding author upon reasonable request.
