# Supplementary material for: Mitsugumin 53 Inhibits Angiogenesis Through Regulating Focal Adhesion Turnover and Tip Cell Formation
Source: J Cell Mol Med. 2025 Feb 24;29(4):e70439. doi: 10.1111/jcmm.70439 (PMC11850094; doi:10.1111/jcmm.70439)
Supplement: Supplementary file 1 — FIGURE S1. Cholesterol is required for the uptake of rhMG53 into endothelial cells. FIGURE S2. Wortmannin does not inhibit the uptake of rhMG53 into endothelial cells. FIGURE S3. rhMG53 induces the increase of intracellular MG53. FIGURE S4. rhMG53 inhibits endothelial cell migration and tube formation. FIGURE S5. The changes of vinculin fluorescence intensity at the edge of the scratch. FIGURE S6. rhMG53 has no effect on the expression of integrins. FIGURE S7. rhMG53 decreases the activation of integrin β1. FIGURE S8. Endothelial cells cultured in 3D collagen gel cultures. [file JCMM-29-e70439-s001.docx]

**
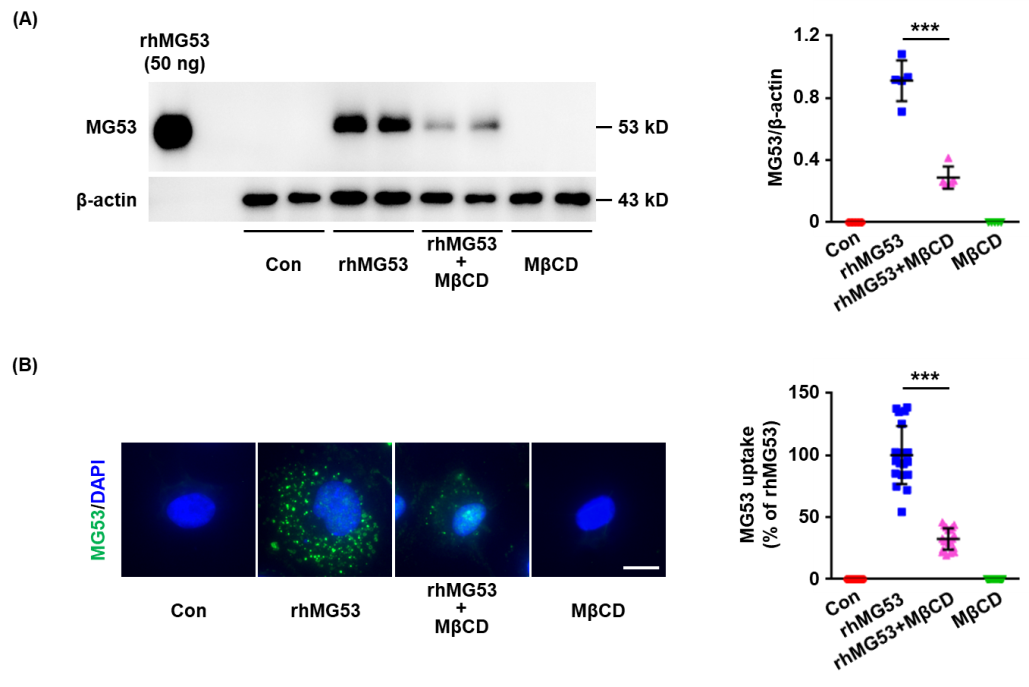
**

**FIGURE S1 Cholesterol is required for the uptake of rhMG53 into endothelial cells.** HUVECs were pre-treated with MβCD (5 mM) for 60 min, followed by stimulation with rhMG53 (20 μg/mL) for 60 min. (A) Cell lysates were harvested and intracellular MG53 was detected by western blotting. Representative images are shown on the left. The densitometric analysis of MG53 normalized to β-actin was performed (n=5 biological replicates). (B) HUVECs were fixed and incubated with a primary anti-MG53 antibody, followed by immunostaining using a FITC-conjugated secondary antibody (green). DAPI-stained nuclei are shown in blue. Representative images from 4 independent experiments are shown. The scale bar is 10 μm. The average fluorescent intensity of MG53 per cell was determined (n=20 fields of view per group, from 4 independent experiments). For the above, data is presented as mean ± SD. ****p* < 0.001 (one-way ANOVA with Tukey multiple comparisons).

**
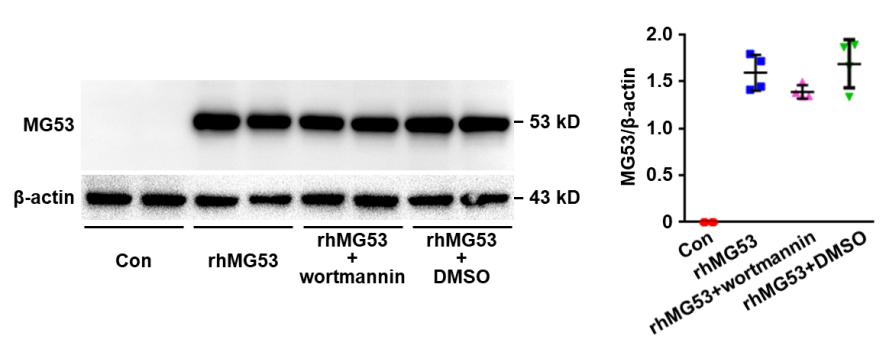
**

**FIGURE S2 Wortmannin does not inhibit the uptake of rhMG53 into endothelial cells.** HUVECs were pre-treated with wortmannin (1 μM) for 60 min, followed by stimulation with rhMG53 (20 μg/mL) for 60 min. Cell lysates were harvested and intracellular MG53 was detected by western blotting. Representative images are shown on the left. The densitometric analysis of MG53 normalized to β-actin was performed (n=4 biological replicates). Data is presented as mean ± SD.

**
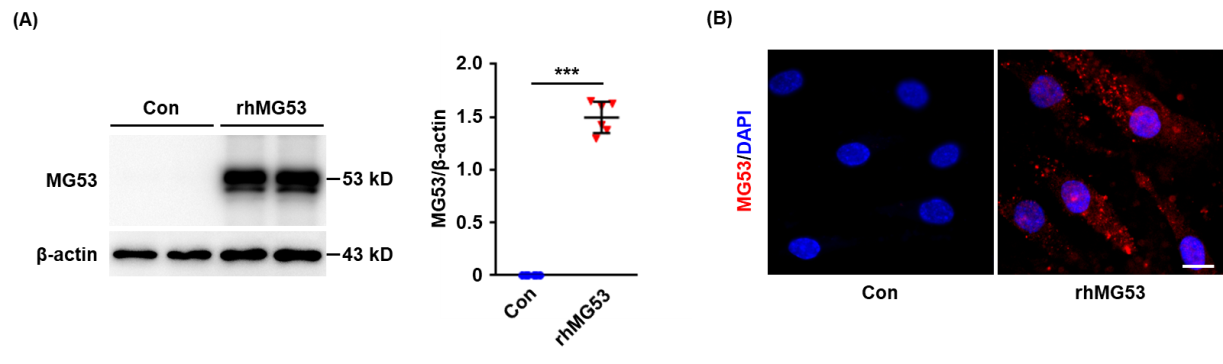
**

**FIGURE S3 rhMG53 induces the increase of intracellular MG53.** HUVECs were incubated for 24 h with either vehicle control or rhMG53 (20 μg/mL). (A) Cell lysates were harvested and intracellular MG53 was detected by western blotting. Representative images are shown on the left. The densitometric analysis of MG53 normalized to β-actin was performed (n=6 biological replicates). Data is presented as mean ± SD. ****p* < 0.001 (two-tailed unpaired Student’s t-test). (B) HUVECs were fixed and incubated with a primary anti-MG53 antibody, followed by immunostaining using an Alexa 594-conjugated secondary antibody (red). DAPI-stained nuclei are shown in blue. Representative images from 4 independent experiments are shown. The scale bar is 10 μm.


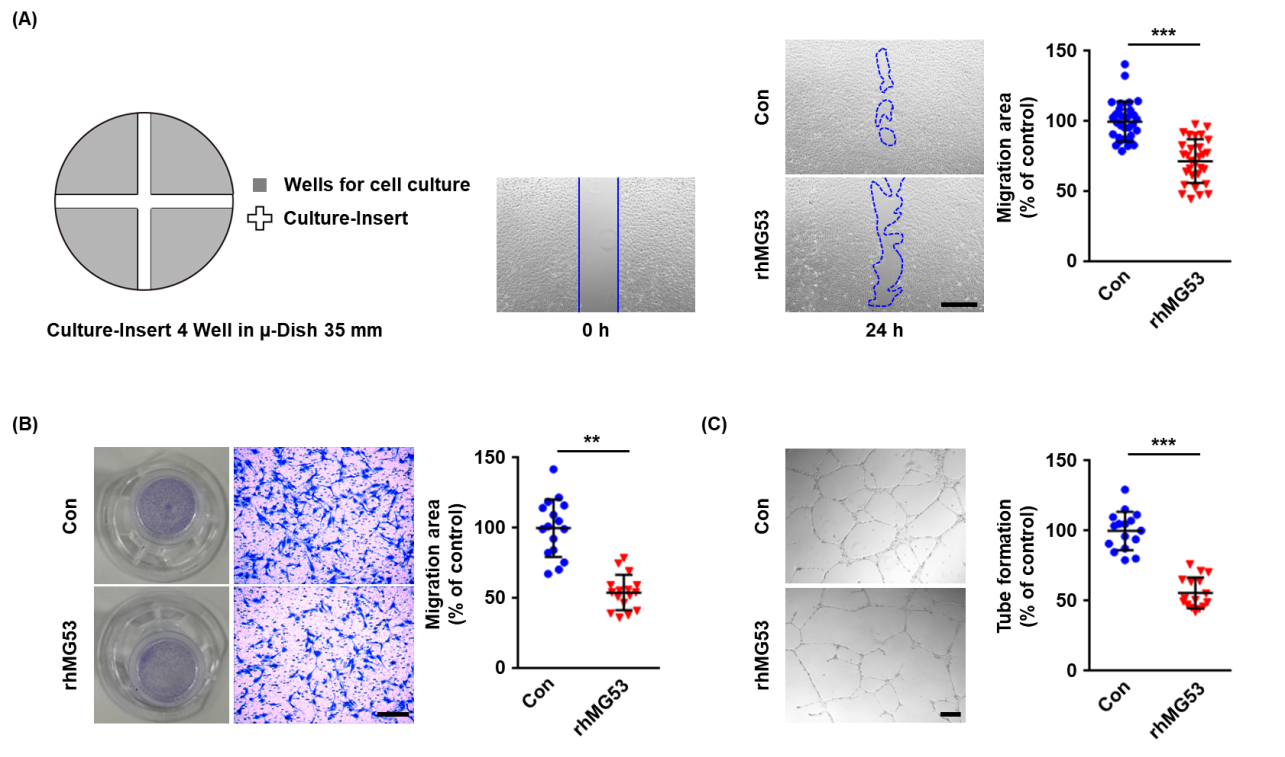


**FIGURE S4 rhMG53 inhibits endothelial cell migration and tube formation.** (A) For performing the wound healing migration assay, HUVECs were seeded in Culture-Insert 4 Well in ibidi μ-Dish. The Culture-Insert 4 well consists of four wells, which are separated by the Culture-Insert and used for cell culture. Cells were allowed to grow to 90% confluence in the wells, followed by incubation with vehicle control or rhMG53 (20 μg/mL) for 24 h. At the end of the incubation, the Culture-Insert was removed and images were acquired to obtain the initial images (0 h). Then the cells were allowed to migrate for another 24 h and the migration area was calculated and quantitatively analyzed (n=30 fields of view per group, from 5 independent experiments). Representative images from 5 independent experiments are shown. The scale bar is 500 μm. (B) HUVECs cultured in six-well plates were exposed to vehicle control or rhMG53 (20 μg/mL) for 24 h. The cells were then detached and added to the upper chambers containing porous filters. After 24 h, the cells were fixed, stained, and the cells that had migrated to the lower chambers were counted. Representative images from 4 independent experiments are shown and quantitative assessment was performed (n=16 fields of view per group, from 4 independent experiments). The scale bar is 200 μm. (C) HUVECs cultured in six-well plates were exposed to vehicle control or rhMG53 (20 μg/mL) for 24 h. The cells were then detached and seeded onto Matrigel for 18 h. Representative images of 4 independent experiments are shown and quantitative assessment was performed (n=16 fields of view per group, from 4 independent experiments). Scale bar is 250 μm. For the above, data are presented as mean ± SD. ****p* < 0.001 (two-tailed unpaired Student’s t-test).


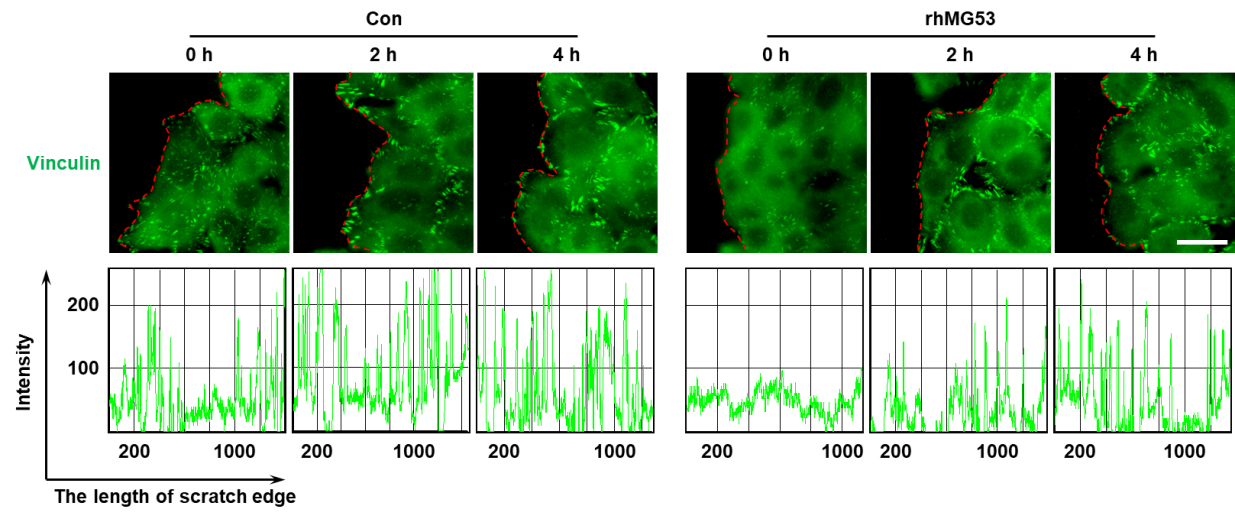


**FIGURE S5 The changes of vinculin fluorescence intensity at the edge of the scratch.** HUVECs grown to 90% confluence were incubated for 24 h with either vehicle control or rhMG53 (20 μg/mL). Afterward, the cells were scratched with a 200 μL pipette tip and allowed to migrate for an additional 0, 2 and 4 h, followed by immunostaining for vinculin (green). The red dotted line indicates the scratch edge and the fluorescence intensity of vinculin along the scratch edge is shown. Representative images from 4 independent experiments are shown. The scale bar is 20 μm.


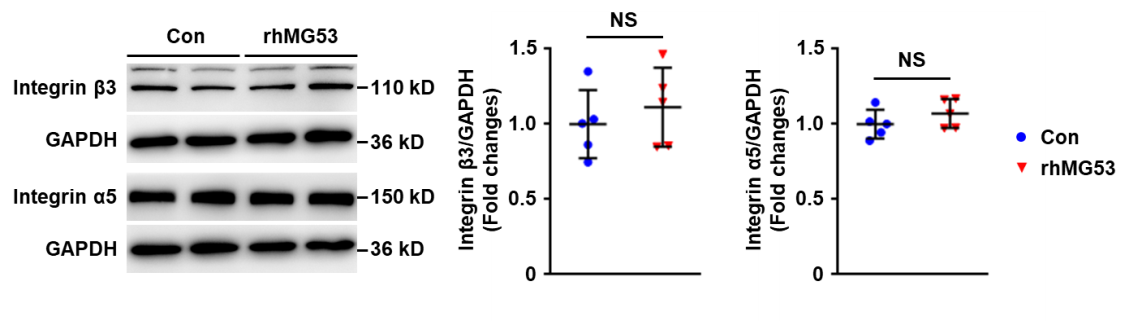


**FIGURE S6 rhMG53 has no effect on the expression of integrins.** HUVECs were incubated for 24 h with either vehicle control or rhMG53 (20 μg/mL) and then the cell lysates were prepared. The expression of integrin β3 and integrin α5 was detected by western blotting. Representative images from 5 independent experiments are shown. The densitometric analysis of integrin β3 and integrin α5 normalized to GAPDH was performed. All data shown is presented as mean ± SD. NS, no significant (two-tailed unpaired Student’s t-test).


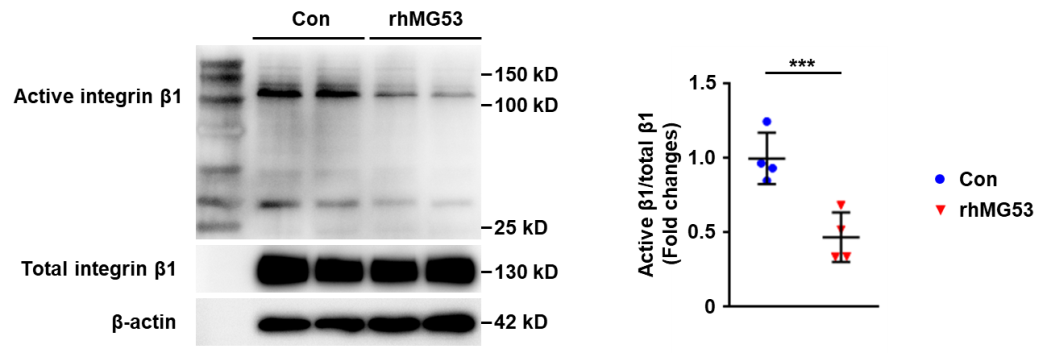


**FIGURE S7 rhMG53 decreases the activation of integrin β1.** HUVECs were incubated for 24 h with either vehicle control or rhMG53 (20 μg/mL) and then the cell lysates were prepared. The expression of active integrin β1 and total integrin β1 was detected by western blotting. Representative images from 4 independent experiments are shown. The densitometric analysis of active integrin β1 normalized to total integrin β1 was performed. All data shown is presented as mean ± SD. ****p* < 0.001 (two-tailed unpaired Student’s t-test).


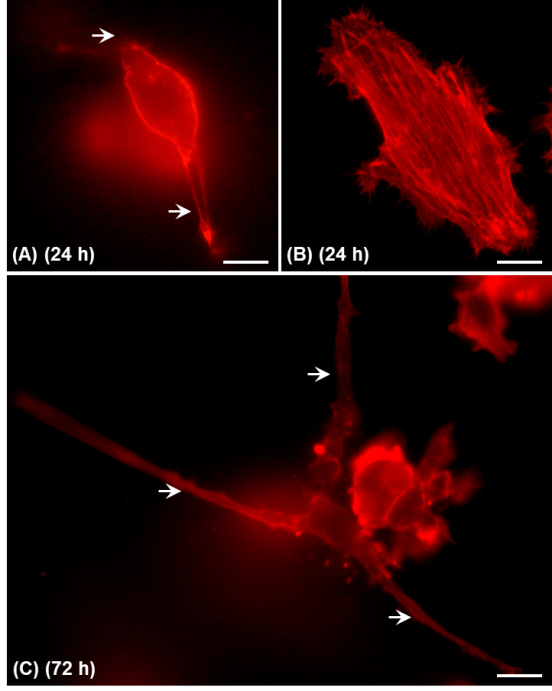


**FIGURE S8 Endothelial cells cultured in 3D collagen gel cultures.** HUVECs were seeded into 3D collagen gel cultures, followed by feeding with media containing reduced serum supplement II (RSII), ascorbic acid and FGF-2 (40 ng/mL) for 24 h or 72 h. The cultures were fixed in 4% paraformaldehyde and stained with rhodamine-conjugated phalloidin. (A) The cell grown in the collagen gel cultures show the features of tip cells with marked filopodial extensions (arrowheads). The scale bar is 5 μm. (B) The cell grown on the bottom of the collagen gel shows 2D cell morphology. The scale bar is 5 μm. (C) Endothelial cell tubulogenesis are shown (arrowheads) after 72 h culture in 3D collagen gel. The scale bar is 5 μm.
